# In Vitro In Silico Screening Strategy and Mechanism of Novel Tyrosinase Inhibitory Peptides from Nacre of *Hyriopsis cumingii*

**DOI:** 10.3390/md22090420

**Published:** 2024-09-15

**Authors:** Haisheng Lin, Fei Li, Jiaao Kang, Shaohe Xie, Xiaoming Qin, Jialong Gao, Zhongqin Chen, Wenhong Cao, Huina Zheng, Wenkui Song

**Affiliations:** 1 Guangdong Provincial Key Laboratory of Aquatic Products Processing and Safety, National Research and Development Branch Center for Shellfish Processing (Zhanjiang), Guangdong Provincial Engineering Technology Research Center of Seafood, Guangdong Province Engineering Laboratory for Marine Biological Products, College of Food Science and Technology, Guangdong Ocean University, Zhanjiang 524088, China; linhs@gdou.edu.cn (H.L.); 2112203055@stu.gdou.edu.cn (F.L.); 202111211412@stu.gdou.edu.cn (J.K.); qinxm@gdou.edu.cn (X.Q.); gaojl@gdou.edu.cn (J.G.); chenzhongqin@gdou.edu.cn (Z.C.); cwenhong@gdou.edu.cn (W.C.); zhenghn@gdou.edu.cn (H.Z.); 2 Shenzhen Institute of Guangdong Ocean University, Shenzhen 518108, China; 3 Collaborative Innovation Center of Seafood Deep Processing, Dalian Polytechnic University, Dalian 116034, China; 4 Guangdong Shaohe Pearl Co., Ltd., Shantou 515041, China; xsh5760288@126.com

**Keywords:** *Hyriopsis cumingii*, molecular docking, B16F10 cells, melanogenesis, antioxidant

## Abstract

For thousands of years, pearl and nacre powders have been important traditional Chinese medicines known for their skin whitening effects. To prepare the enzymatic hydrolysates of *Hyriopsis cumingii* nacre powder (NP-HCH), complex enzymatic hydrolysis by pineapple protease and of neutral protease was carried out after the powder was pre-treated with a high-temperature and high-pressure method. The peptides were identified using LC-MS/MS and picked out through molecular docking and molecular dynamics simulations. Subsequently, the tyrosinase inhibitory and antioxidant properties of novel tyrosinase inhibitory peptides were investigated in vitro. In addition, the enzymatic activity of tyrosinase in B16F10 cells as well as melanin content and antioxidant enzyme levels were also examined. The results showed that a tyosinase inhibitory peptide (Tyr-Pro-Asn-Pro-Tyr, YPNPY) with an efficient IC_50_ value of 0.545 ± 0.028 mM was identified. The in vitro interaction results showed that YPNPY is a reversible competitive inhibitor of tyrosinase, suggesting that it binds to the free enzyme. The B16F10 cell whitening test revealed that YPNPY can reduce the melanin content of B16F10 cells by directly inhibiting the activity of intracellular tyrosinase. Additionally, it indirectly affects melanin production by acting as an antioxidant. These results suggest that YPNPY could be widely used as a tyrosinase inhibitor in whitening foods and drugs.

## 1. Introduction

*Hyriopsis cumingii* is the most significant freshwater pearl-producing mussel in China, and its primary role is the culture of pearls, which have significant economic value [1,2]. In traditional Chinese medicine, the pearl shell is believed to have a calming effect on the liver and kidneys, which, in turn, calms the mind, cleanses the liver, and brightens the eyes. Shell and pearl have a similar structure and composition of materials. They are both formed by the secretion of an organic matrix, which is regulated by the formation of biominerals [3,4]. The shell structure can be divided into three distinct layers: the nacre layer, the prismatic shell, and the cuticle. The nacre layer is secreted by the outer mantle tissue and consists of a lustrous layer of nacreous material. Over time, this material gradually envelops the foreign bodies and eventually forms a pearl. The pearl layer of the shell consists of approximately 95% calcium carbonate and 5% organic matrix [5,6]. Nacre powder contains a variety of amino acids, including hydrophobic and aromatic types; the available studies have demonstrated that peptides capable of inhibiting tyrosinase are typically composed of these amino acids. Consequently, peptides derived from pearl layers have emerged as a noteworthy class of tyrosinase-inhibiting peptides. Antibacterial [7], anti-photoaging [8], and antioxidant [9] activities are some of the functional activities of pearl powder found in modern medicine; nacre powder is very similar to pearl powder, but their whitening activity remains worthy of exploration, as the ingredients and factors contributing to the whitening effect are still not clear.

The color of human skin and hair is largely determined by the distribution of melanin [10]. Melanin, classified as eumelanin and pheomelanin, is a phenolic polymer widely found in plants and animals [11]. It inhibits the stimulation and damage to skin cells caused by external factors such as ultraviolet rays and free radicals. When cells are exposed to excessive UV light, tyrosine in melanocytes in the basal layer of the epidermis is oxidized by tyrosinase to DOPA. DOPA is then further oxidized to dopaquinone, a highly reactive molecule, that can produce polymers or brown pigments when reacting with amino acids or proteins, ultimately leading to melanin production [12]. The excessive accumulation of melanin on the skin’s surface can result in various skin disorders, such as dark spots and brown spots, accelerate skin aging, and potentially contribute to the development of serious diseases like melanoma. Tyrosinase, a copper-containing metal oxidase, is particularly important because it is closely related to the process of melanin production. By inhibiting tyrosinase, it is possible to regulate melanin production, thereby enabling the skin to achieve a lighter tone or alleviate skin conditions such as melanoma. There are two main ways to inhibit tyrosinase: first, tyrosinase inhibitors directly inhibit the active site of tyrosinase and interact at the active site. Second, they can influence tyrosinase content indirectly by modulating the antioxidant enzyme system and antioxidant metabolites [13,14]. Effective whitening ingredients that are proven and widely available on the market, such as Kojic acid and its derivatives and arbutin, have a stronger inhibitory effect on tyrosinase [15,16]. However, they are also prone to some side effects [17]. The effects vary from person to person and can lead to allergies, skin rashes, dermatitis, and other side effects [18]. Whitening preparations that are safe and stable for the human body are receiving increasing attention and represent the future direction of the cosmetic industry’s efforts [19]. Humans produce a large number of by-products when extracting pearls, including a significant amount of mussels and shell meat [20]. Previous studies have concentrated on the mechanism of coloration and the process of biomineralization in sail mussel pearls. Jin et al. isolated a new matrix protein (myosin) from the *Hyriopsis cumingii* mussel and found that it is involved in the regulation of crystal morphology during the formation of the bead layer and the induction of polycrystal formation [6]. Qiao et al. found that HCPS from *Hyriopsis cumingii* increased the antioxidant enzyme activities and total antioxidant capacity in a dose-dependent manner [21]. Previous studies have shown that peptides extracted from pearl shells and their tissues have demonstrated a variety of biological activities such as antioxidant [9], antimicrobial [22], and neuroprotective effects [23]. However, limited research has been conducted on potential whitening peptides found within the pearl shells.

In this study, the pearl layer powder of *Hyriopsis cumingii* was pre-treated with high temperature and pressure. To degrade the insoluble proteins in the pearl layer of *Hyriopsis cumingii*, a combination of neutral protease and pineapple protease was used for enzymatic hydrolysis. Potential tyrosinase inhibitory peptides were identified and screened for molecular docking using LC-MS/MS. The solid-phase synthesis of peptide sequences, their mechanisms of tyrosinase inhibition, and their antioxidant capabilities were investigated in relation to the in vitro tyrosinase activity, the viability of B16F10 cells, and intracellular melanin-related indicators. The study utilizes the nacre of *Hyriopsis cumingii* to identify effective tyrosinase-inhibitory peptides, with the aim of developing natural and non-toxic medicinal cosmetics. This study contributes to the development of new cosmetic ingredients and natural food-based functional foods.

## 2. Results and Discussion

### 2.1. Inhibition of Tyrosinase and Antioxidant Activity of NP-HCH

**Figure 1:**
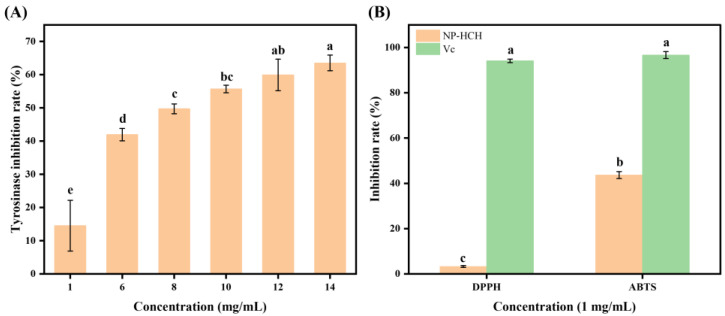
(**A**) Tyrosinase inhibitory activity of enzymatic hydrolysis product NP-HCH. (**B**) Antioxidant activity of enzymatic hydrolysis product NP-HCH. Different letters indicate that there are significant differences in data (*p* < 0.05).

A preliminary study was carried out to evaluate the tyrosinase activity and antioxidant properties of the enzymatic hydrolysis product derived from nacre powder, following treatment under high temperature and pressure, as well as complex enzymatic digestion. The results, detailed in Figure 1, demonstrate that the nacre peptide digest exhibits a notable inhibitory effect on tyrosinase activity with an IC_50_ value of (7.596 ± 0.808) mg/mL and possesses antioxidant capabilities. At a concentration of 1 mg/mL, the enzymatic digest significantly inhibited ABTS radicals more than DPPH radicals and tyrosinase (*p* < 0.05). These findings indicate the potential for developing peptides that inhibit tyrosinase.

### 2.2. Identification and Molecular Docking

The mass spectrometry data were analyzed using the software PEAKS Studio 8.5. The identification results were compared with the UniProt database. For efficient screening, peptides with amino acid residues < 10 and score > 20 were selected for docking with tyrosinase (2Y9X) [24].

**Figure 2:**
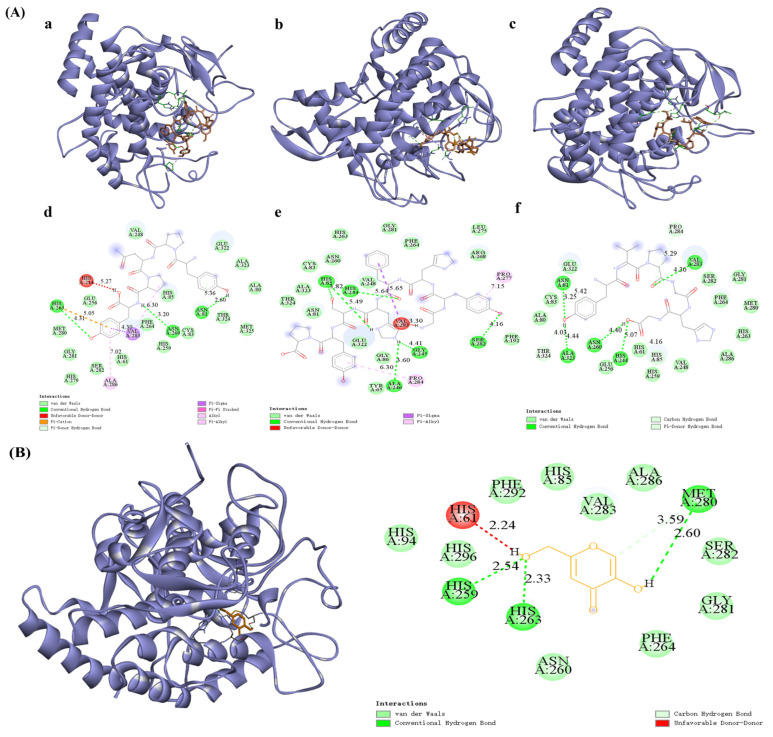
(**A**) The 3D and 2D visualizations of molecular docking of YPNPY, GYHFHSYP, and YVPGHG with tyrosinase (2Y9X). (**a**–**f**). (**B**) The 3D and 2D visualizations of molecular docking of Kojic acid.

Molecular docking is a method that simulates interactions between small molecules and proteins using computer software and can predict binding affinities [25]. The molecular docking process is carried out by AutoDock Vina on processed peptides with tyrosinase as the protein receptor. The lower the binding energy between the molecule and the protein, the better the peptide–protein affinity [26]. The tyrosinase inhibitory activity of peptides is closely related to their amino acid composition, such as hydrophobic amino acids, aromatic amino acids, and polar amino acids, which play a crucial role in protein–ligand interactions [27]. Additionally, the composition of amino acids influences the water solubility of peptides. The smaller the GRAVY value is, the more hydrophilic the peptide is. Generally speaking, peptides that contain hydrophobic amino acids and aromatic amino acids have a greater ability to inhibit tyrosinase. A smaller docking binding energy also indicates that the peptide binds more stably to tyrosinase. In cases where the amino acid composition is approximately the same, we prefer short peptides. As shown in Table 1 and Figure 2, considering factors such as hydrophobicity, amino acid composition, peptide length, and free binding energies, three peptides—GYHFHSYP, YPNPY, and YVPGHG—were chosen for further investigation. The predicted binding energies for these peptides were −10.7 kcal/mol for GYHFHSYP, −10.4 kcal/mol for YPNPY, and −9.4 kcal/mol for YVPGHG, indicating that they may possess theoretical inhibitory effects on tyrosinase. The peptide identification results show that the relative content of YPNPY is 4.53%. Docking binding was slightly lower than the results for peptides extracted from *Atrina pectinata* mantle, where a lower binding energy indicates a more favorable binding effect [28]. To visually demonstrate how the peptide interacts with tyrosinase, a visual analysis was conducted using the Discovery Studio 2019 client tool. As shown in Table 2, the peptide YPNPY establishes five hydrogen bonds with tyrosinase at the residues HIS85, ALA246, GLY245, SER282, and HIS244. Meanwhile, GYHFHSYP forms three hydrogen bonds with tyrosinase’s HIS263, ASN260, and ASN81, and YVPGHG creates five hydrogen bonds with tyrosinase, along with several hydrophobic interactions. Compared to the positive control compound Kojic acid, peptide YPNPY forms hydrogen bonds with HIS263 of tyrosinase, just like Kojic acid. The findings indicate that the peptide interacts with certain residues close to the active site pocket of tyrosinase, including HIS263, ASN260, and VAL283.

### 2.3. Molecular Dynamics Simulation

**Figure 3:**
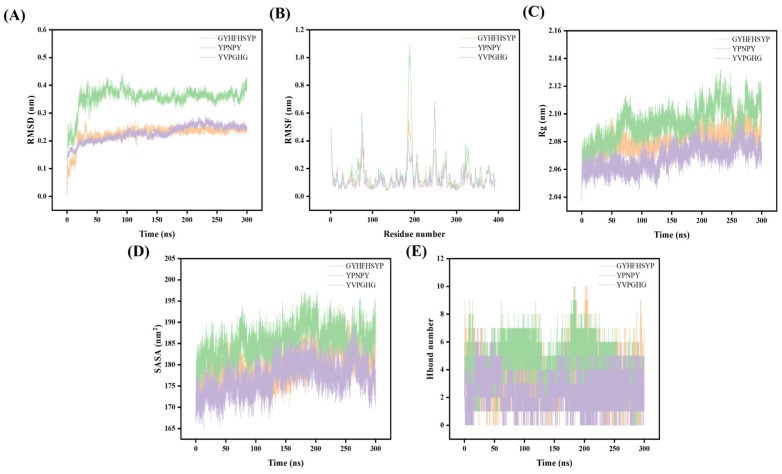
Molecular dynamics results of tyrosinase with YPNPY, GYHFHSYP, and YVPGHG: (**A**) RMSD; (**B**) RMSF; (**C**) Rg; (**D**) SASA; and (**E**) Hbond number.

The stability and binding affinity of tyrosinase with three small molecules were systematically investigated using GROMACS 2022.3 software, employing the docked three-complex system as the foundational structure. To obtain stable complex systems, a 300 ns molecular dynamics (MD) simulation was performed, allowing a comparative analysis of the three small molecules at the molecular level. The trajectory data generated from the 300 ns simulation were analyzed to extract the kinetic properties of the three complexes. Key metrics such as the root mean square deviation (RMSD), radius of gyration (Rg), solvent accessible surface area (SASA), and root mean square fluctuation (RMSF) were employed to assess each system within the MD study, with the results presented in Figure 3.

The root mean square deviation (RMSD) serves as a quantitative measure for evaluating structural alterations in proteins, specifically by calculating the average deviation between the conformation of a protein–ligand complex and its original conformation at a specified time point [29]. The results indicated that the RMSD values remained within a narrow range (less than 1), suggesting that the interaction between proteins and small molecules exhibited a degree of stability. Furthermore, the RMSD trajectories for the protein–small molecule complexes were relatively smooth. Notably, the equilibrium velocity of tyrosinase in complex with GYHFHSYP was observed to be greater than that of tyrosinase in complex with GYHFHSYP after 30 nanoseconds of simulation. In contrast, the RMSD profile for tyrosinase with YVPGHG remained stable, while the RMSD for tyrosinase with YPNPY exhibited a gradual increase after 60 nanoseconds, indicating that this complex experienced structural changes and began to exhibit instability. The root mean square fluctuation (RMSF) serves as a quantitative measure for evaluating the dynamic behavior of proteins by averaging the variations in atomic positions over time [30]. This metric is instrumental in characterizing the flexibility and movement intensity of amino acids within a protein during simulations. The findings indicate that the RMSF values are notably higher in the binding region, specifically between 180–190, with the fluctuation of tyrosinase in conjunction with YPNPY reaching a peak of 1 nm. The minimal fluctuation indicates that these atoms create stable complexes with tyrosinase because of robust intermolecular interactions, restricting their movement in molecular dynamics simulations. Gyrate is a metric used to assess the overall compactness of a protein by characterizing the distribution of system atoms along a specific axis [31]. In this simulation, we calculated the Gyrate between the protein and small molecules and generated a Gyrate graph. In the binding of the three peptides to tyrosinase, the Gyrate exhibited by tyrosinase with YVPGHG fluctuates within the smallest range compared to the other two peptides. Conversely, the Gyrate value of tyrosinase with GYHFHSYP shows a pattern of initial increase followed by a decrease, suggesting that the binding of the small molecules has increased the protein molecule’s compactness. The Gyrate value with YPNPY exhibited a similar trend to the RMSD value, initially increasing and then decreasing after 60 ns, implying that the overall protein change during the binding process of YPNPY with tyrosinase was more significant compared to the other two peptidase complexes. The solvent accessible surface area (SASA) serves as a metric for evaluating the surface area of proteins, specifically the area accessible to solvents in biomolecules, and it is instrumental in predicting the degree of conformational alterations that occur during binding events. In the present simulation, we computed the SASA values for interactions between proteins and small molecules, subsequently generating SASA graphs for analysis. The findings reveal that the curves representing the three peptide–enzyme complexes exhibit consistent fluctuations in amplitude. Notably, the complex formed between tyrosinase and YPNPY demonstrates the highest SASA, suggesting that interactions between proteins and small molecules significantly influence the characterization and stability of protein molecular surfaces. Hydrogen bonding serves as a criterion for evaluating the interactions between proteins and small molecules. The findings indicated that a significant number of hydrogen bonds were established between proteins and small molecules throughout the simulation [32]. These hydrogen bonds primarily represent internal interactions involving critical residues of tyrosinase and peptides, as observed in the molecular docking outcomes.

An analysis of the results pertaining to the RMSD, RMSF, Gyrate, SASA, and hydrogen bond counts for the three peptide–enzyme complexes indicates that YPNPY has a more pronounced structural influence on tyrosinase. Furthermore, it is suggested that YPNPY may induce a disassembling effect on the enzyme. Consequently, it can be inferred that YPNPY likely exerts a comparatively greater effect on the activity of tyrosinase than GYHFHSYP in conjunction with YVPGHG.

### 2.4. Tyrosinase Inhibitory Activity and Antioxidant Capacity of Synthetic Peptides

In terms of amino acid composition, the peptide YPNPY comprises two repetitive amino acid residues containing benzene rings, specifically Tyr, along with two hydrophobic amino acids, Pro. Research indicates that such repetitive amino acid sequences can enhance the inhibition of tyrosinase. Similarly, the peptides GYHFHSYP and YVPGHG also contain polar amino acids, benzene-ring-containing residues, and hydrophobic amino acids. It has been documented that the presence of hydrophobic amino acids, aromatic amino acids, and polar amino acids contributes to the inhibition of tyrosinase activity [24]. As illustrated in Table 3, the peptides YPNPY, GYHFHSYP, and YVPGHG, which are derived from *Hyriopsis cumingii*, exhibit varying degrees of inhibitory efficacy against tyrosinase, with Kojic acid serving as a positive control. Notably, YPNPY demonstrated a significant inhibitory effect, with an IC_50_ value of 0.545 ± 0.028 mM, corroborating the findings obtained through molecular docking and molecular dynamics simulations.

**Figure 4:**
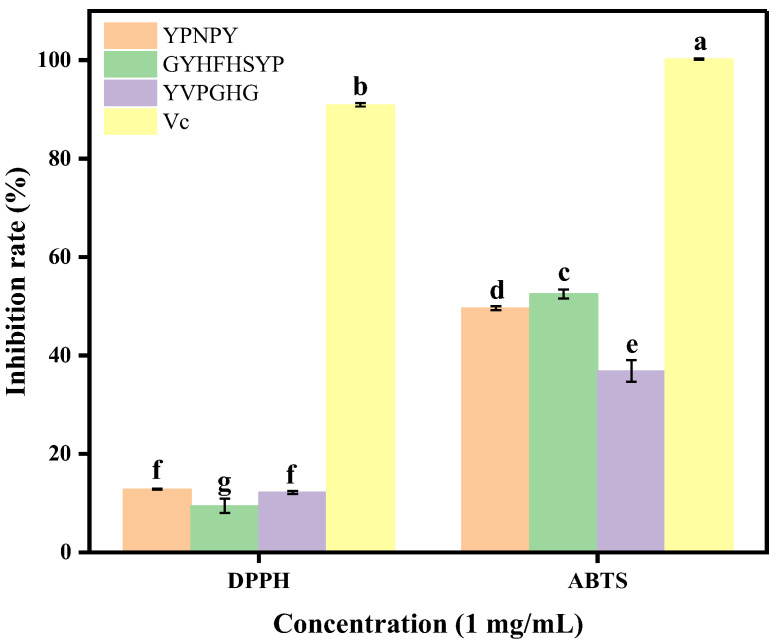
Scavenging capacity of peptides for DPPH and ABTS free radicals. Different letters indicate that there are significant differences in data (*p* < 0.05).

The data presented in Figure 4 indicate that the synthetic peptides YPNPY, GYHFHSYP, and YVPGHG, derived from *Hyriopsis cumingii*, exhibited varying levels of radical scavenging activity against DPPH and ABTS radicals. The scavenging effect of the three peptides on ABTS radicals differed significantly from that on DPPH radicals (*p* < 0.05). The scavenging effect of the three peptides on DPPH radicals remained consistent at a concentration of 1 mg/mL (*p* > 0.05), while the scavenging ability of the three peptides on ABTS radicals was ranked as follows: GYHFHSYP > YPNPY > YVPGHG (*p* < 0.05).

**Figure 5:**
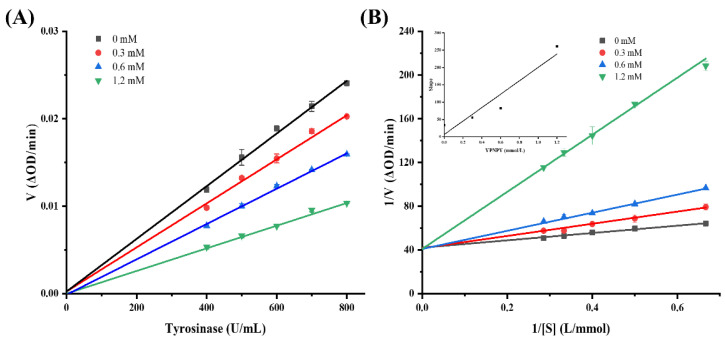
(**A**) Plots of enzymatic reaction rate versus tyrosinase concentration. (**B**) Plots of Lineweaver–Burk; the secondary plots of slope and Y-intercept versus concentration of YPNPY are shown in the inset.

From Figure 5, it can be seen that the slope of the concentration–rate curve of tyrosinase passes through the origin of the coordinate axis, indicating that the inhibition of tyrosinase by YPNPY can be considered a reversible reaction. The type of inhibition of tyrosinase by the synthetic peptide YPNPY was further determined by creating a Lineweaver–Burk double reciprocal plot of the enzymatic reaction rate against the concentration of the substrate (L-tyrosine) [33]. The intercept of the straight line with the *Y*-axis in the double reciprocal plot represents 1/Vmax, while the intercept with the *X*-axis is −1/Km. As depicted in the figure, the reciprocal of the enzymatic reaction rate exhibited a linear relationship with the reciprocal of the substrate concentration as the concentrations of tyrosinase, YPNPY (0, 0.3, 0.6, and 1.2 mM, respectively), and the substrate L-tyrosine were varied. The slope increased and intersected the *Y*-axis with the concentration of the synthetic peptide. With an increase in the concentration of the synthetic peptide, the slope of the straight line grew larger and intersected the *Y*-axis at a specific point, indicating that YPNPY acts as a competitive inhibitor of tyrosinase [34].

**Figure 6:**
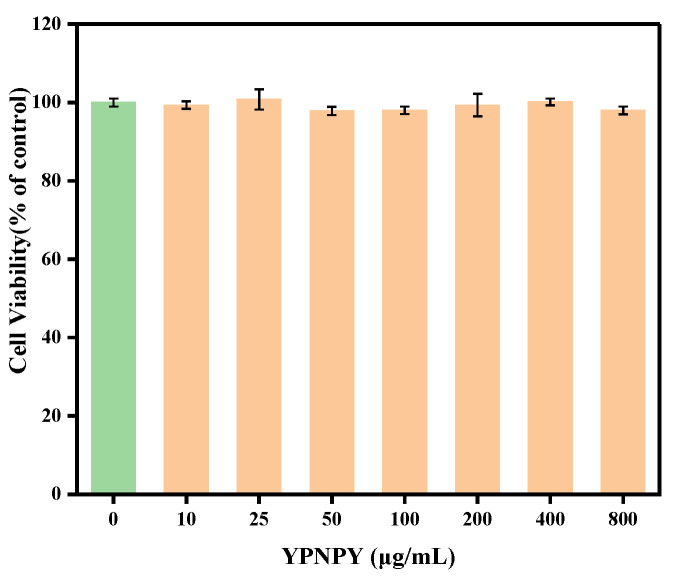
Effects of YPNPY on cell viability.

As depicted in Figure 6, in order to investigate the effect of YPNPY on the viability of B16F10 cells, we utilized peptide concentrations ranging from 10 to 800 μg/mL for the study; 0 μg/mL is the blank group. It can be seen that, after 24 h of YPNPY treatment, the viability of the sample treatment group on B16F10 cells is close to 100%; there was no significant difference in the viability of B16F10 cells treated with the synthetic peptide YPNPY within the concentration range of 10–800 μg/mL (*p* > 0.05) compared to the control group. This suggests that the synthetic peptide YPNPY did not exhibit any cytotoxic effects; this result is consistent with the findings of Huang et al. on B16F10 cells using peptides extracted from pearl shell meat [24]. Subsequently, concentrations of 10, 25, and 50 μg/mL were chosen for further experiments.

**Figure 7:**
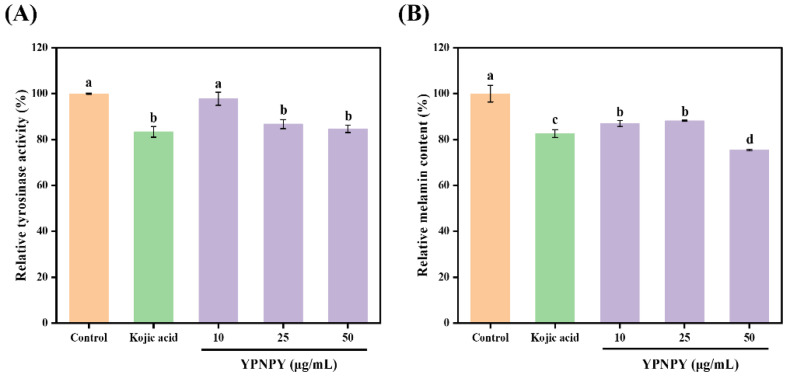
(**A**) Effects of YPNPY and Kojic acid on tyrosinase activity in B16F10 cells. (**B**) Effects of YPNPY and Kojic acid on melanin production in B16F10 cells. c (Kojic acid) = 50 μg/mL. Different letters indicate that there are significant differences in data (*p* < 0.05).

As can be seen in Figure 7, in comparison to the control group, YPNPY notably suppressed the tyrosinase activity of B16F10 cells within the concentration range of 10–50 μg/mL (*p* < 0.05). The intracellular melanin content was also assessed within the same concentration range (*p* < 0.05). This result is similar to the findings of Yu et al., who studied the inhibition of tyrosinase enzyme activity and melanin content in B16F10 cells [35]. At the highest treatment concentration, the intracellular tyrosinase activity and melanin content decreased by 15.34% and 24.53%, respectively. In contrast, treatment with Kojic acid led to a 16.64% reduction in tyrosinase activity and a 17.39% decrease in melanin content.

**Figure 8:**
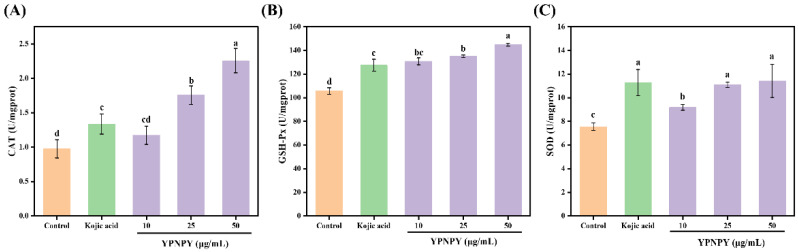
Effect of YPNPY on the intracellular antioxidant capacity in B16F10 cells: (**A**) CAT; (**B**) GSH-Px; and (**C**) SOD. Concentration of Kojic acid = 50 μg/mL. Different letters indicate that there are significant differences in data (*p* < 0.05).

SOD, CAT, and GSH-Px are antioxidant enzymes that collaborate to manage antioxidant functions and shield cells from damage caused by oxidative stress [36]. As shown in Figure 8, the data indicate that the activities of GSH-Px and SOD enzymes are dependent on the concentration of YPNPY. At the highest treatment concentration, the activities of CAT, GSH-Px, and SOD increased by 112.38%, 37.41%, and 70.19%, respectively, while, under Kojic acid treatment, they rose by 32.04%, 25.56%, and 68.81%, respectively. At a concentration of 50 μg/mL, CAT and GSH-Px activities were significantly higher (*p* < 0.05) compared to Kojic acid. At a concentration of 50 μg/mL, there was no significant difference in SOD activity between YPNPY and Kojic acid (*p* > 0.05). Therefore, YPNPY demonstrated a protective effect on the antioxidant system by enhancing the activity of antioxidant enzymes in B16F10 cells, which helped prevent melanin production. Compared to the pentapeptide studied by Hu et al., the three antioxidant enzymes displayed a similar trend following treatment with the peptide, which enhances the activity of these enzymes. The increase in antioxidant enzyme activity may inhibit the generation of reactive oxygen species (ROS), thereby achieving an antioxidant effect.

## 3. Materials and Methods

### 3.1. Materials

Nacre powder of the *Hyriopsis cumingii* (the bivalve class, family Unionidae, and genus Pseudanodonta belong to the group of bivalve mollusks) was purchased from Guangdong Shaohe Pearl Co., Ltd. (Shantou, China). Tyrosinase (from Mushroom, 500 U/mg) and PBS buffer were purchased from Shanghai Yuanye Biotechnology Co., Ltd. (Shanghai, China). L-tyrosine was obtained from Shanghai Macklin Biochemical Technology Co., Ltd. (Shanghai, China). Fetal bovine serum (FBS) was purchased from Serana Europe GmbH (Dorfstrasse 17A, Pessin, Brandenburg, Germany), and the penicillin–streptomycin mixture was purchased from Beijing Soleberg Biotechnology Co., Ltd. (Beijing, China). DMEM/F-12 medium and trypsin-EDTA digest were purchased from Thermo Fisher Scientific (Shanghai, China).

### 3.2. Preparation of Enzymatic Digests of Nacre Peptides

First, 50 g of nacre powder from *Hyriopsis cumingii* was dissolved in 250 mL (*v*:*w* = 5:1) of water and heated at high temperature (121 °C) and pressure for 20 min. Then, 0.1250 g of pineapple protease and 0.2500 g of neutral protease were added, and the enzyme digestion was conducted at a temperature of 50 °C for 4 h. Finally, the mixture was heated at 100 °C for 20 min. The supernatant was centrifuged at 8000 rpm for 20 min at 4 °C to obtain the pearl peptide solution after vacuum concentration.

### 3.3. Tyrosinase Inhibitory Activity and Antioxidant Activity Assay of Enzyme Digests

The methodology employed in this study was adapted from the work of Yu [37], with minor modifications. A solution of L-tyrosine at a concentration of 0.5 mg/mL, along with the sample solution and PBS buffer, was sequentially introduced into a 96-well plate. The mixture was thoroughly homogenized and subsequently incubated at 37 °C for 10 min. Following this incubation, 20 μL of tyrosinase solution (500 U/mL) was added to each well, and the plate was promptly placed in an enzyme marker for a reaction period of (10 min ± 5 s) at 37 °C.

(1) $$Tyrosinase\;inhibition\;rate\%=\left(1-\frac{A_{4}-A_{3}}{A_{2}-A_{1}}\right)\times 100\%$$

where A_4_ is the absorption value of the reaction well, A_3_ is the absorption value of the Sample Background well, A_2_ is the absorption value of the Solvent reaction well, A_1_ is the absorption value of the Solvent base well.

Use a concentration of 1 mg/mL of the enzymatic hydrolysate to assess the free radical scavenging rate. DPPH and ABTS free radical scavenging capacity assays were conducted using kits from Grace Biotechnology Co., Ltd. (Suzhou, Jiangsu, China). The subsequent determination of the inhibitory rate of peptide tyrosinase synthesis and the antioxidant measurement methods refer to this step.

### 3.4. Protein Sequence Identification

The amino acid sequence was identified by using the Bio-Tech Pack Technology Co. (Beijing, China). The nacre peptides were reduced and alkylated separately as samples, with an Easy-nLC 1200 system coupled with a Q Exactive™ Hybrid Quadrupole-Orbitrap™ Mass Spectrometer (Thermo Fisher Scientific, Waltham, MA, USA) with an ESI nanospray source. Then, they were analyzed by liquid chromatography–mass spectrometry (LC-MS/MS) to generate a raw file of the mass spectrometry results. The mass spectrometry data were analyzed using the software PEAKS Studio 8.5, and the identification results were compared with the UniProt database.

### 3.5. Molecular Docking Studies

Referring to the method of Wang [28], ligands and proteins required for molecular docking were prepared using AutoDock Vina 1.1.2 software. Tyrosinase (PDB: 2Y9X) was utilized as the receptor, and the peptides identified after screening were used as ligands. Peptides were designed using ChemDraw and energy-minimized using Chem3D. The crystal structure of the target protein was obtained from the PDB database (https://www.rcsb.org/structure/2Y9X, accessed on 8 June 2024). Autodock Vina software was used to perform molecular docking with default parameters to calculate the binding energy of the peptide sequence to the enzyme. The binding free energy (kcal/mol) of the target structure represents the binding ability of the two molecules, and the lower the binding free energy, the more stable the binding between the ligand and the receptor. Pymol (https://pymol.org/2/, accessed on 16 June 2024) was used for visual analysis, while Discovery Studio 2019 was utilized for further exploration. Discovery Studio 2019 (https://www.3ds.com/products/biovia/discovery-studio, accessed on 16 June 2024) was used for visual analysis of 2D images. Tyrosinase docking centers were X = −10.09, Y = −28.03, and Z = −43.14.

### 3.6. Molecular Dynamics Simulations

The system used in this experiment is a protein–small molecule complex containing a protein molecule and a small molecule. We utilized the Gromacs 2022.3 software to conduct the simulations [38,39]. The process began with molecular preparation, involving the import of the structure files of the protein and the small molecule into Gromacs. Subsequently, we generated the topology files and simulation boxes using the pdb2gmx and gmx editconf commands. The structures of proteins and small molecules were then minimized for simulation through the gmx grompp and gmx mdrun commands. For the molecular dynamics simulation phase, we conducted simulations of proteins and small molecules for up to 300 ns using the gmx grompp and gmx mdrun commands. We recorded the conformational information throughout the simulation period and calculated metrics such as RMSD, RMSF, Gyrate, SASA, and HBond for proteins and small molecules using the gmx rms command. The findings were presented and analyzed by creating graphs and statistical tables.

### 3.7. Peptides Synthesis

Three peptides, Tyr-Pro-Asn-Pro-Tyr (YPNPY), Gly-Tyr-His-Phe-His-Ser-Tyr-Pro (GYHFHSYP), and Tyr-Val-Pro-Gly-His-Gly (YVPGHG), each with a purity above 98% (*w*/*w*), were obtained through solid-phase synthesis by Aminolink Biotechnology Co., Ltd. (Shanghai, China). The synthetic peptides purity was detected by HPLC and the peptide sequences were identified by ESI-MS spectroscopy.

### 3.8. Enzyme Inhibition Kinetic Assay

In this study, the concentration of L-tyrosine was maintained at a constant level of 0.5 mg/mL, while the concentrations of the synthetic peptide and tyrosinase were systematically varied. The optical density (OD_475_) absorbance values were continuously recorded at a temperature of 37 °C following the mixing of the components. The enzyme concentration was represented on the horizontal axis, with the corresponding rate of absorbance change depicted on the vertical axis. Additionally, under conditions where the peptide and substrate concentrations were altered while the tyrosinase solution remained constant, the reciprocal of the substrate concentration was plotted on the horizontal axis, and the reciprocal of the rate of absorbance change was plotted on the vertical axis.

### 3.9. Cytotoxicity of YPNPY to B16F10 Cells

B16F10 cells were purchased from Hunan FengHui Biotechnology Co., Ltd. (Changsha, Hunan, China). Cell cultures of B16F10, specifically from generations 10 to 25, were maintained in a medium consisting of DMEM supplemented with 10% fetal bovine serum and 1% antibiotic–antifungal solution, under conditions of 5% CO_2_ at 37 °C.

Cell viability was evaluated utilizing the Cell Counting Kit-8 (CCK-8) assay. During the logarithmic growth phase, 100 µL of cell suspension, containing 3000 cells per well, was transferred into 96-well plates. Following a 24 h incubation period at 37 °C, the supernatant was discarded. Wells containing 100 µL of medium were designated as blank controls. Viability assessments were conducted 48 h post-treatment of the samples.

### 3.10. Effect of YPNPY on Melanin and Tyrosinase Synthesis in B16F10 Melanoma Cells

Cells in the logarithmic growth phase were cultured at a density of 4 × 10^5^ cells per well in 6-well plates. Following a 24 h incubation period, the culture supernatant was discarded. Experimental groups were established, including a control group, a positive control group treated with Kojic acid at a concentration of 50 µg/mL [40], and peptide-treated groups at concentrations of 10, 25, and 50 µg/mL. After an additional 24 h of incubation, the cells were lysed, and the resulting cell lysates were subjected to centrifugation at 12,000 rpm for 10 min. The reaction mixture was prepared by combining 50 µL of the cell lysate supernatant, 50 µL of 1 mg/mL L-DOPA, and 50 µL of PBS buffer. This mixture was incubated at 37 °C for 1 h. Subsequently, the level of dopamine formation was quantified spectrophotometrically at a wavelength of 475 nm. The activity of tyrosinase was reported as a percentage relative to the blank control [41].

The cell culture stage for melanin content determination was as follows: The cells were washed twice with PBS buffer after 48 h of peptide treatment. Trypsin digestion was performed, and the cells were collected. The lower precipitate was removed by centrifugation for 5 min and the precipitate was lysed by dissolving it in 1 mol/L NaOH containing 10% DMSO for 1 h at 80 °C. Subsequently, the cell lysate was centrifuged at 12,000 rpm for 10 min. Then, 200 µL of the supernatant was transferred to a 96-well plate, and the absorbance was measured at 405 nm. The melanin content was then expressed as a percentage of the control group.

### 3.11. Effect of YPNPY on Antioxidant Enzyme Activity in B16F10 Cells

Cell culture and treatment methods were the same as described in Section 3.10. The activities of catalase (CAT), glutathione peroxidase (GSH-Px), and superoxide dismutase (SOD) were determined using the assay kit from Nanjing Jianjian Bioengineering Research Institute (Nanjing, China) [42].

### 3.12. Statistical Analysis

All data are expressed as the mean ± standard deviation (SD) of at least three different experiments. Multiple group comparisons were conducted using one-way analysis of variance (ANOVA) and Duncan’s multiple-range test in IBM SPSS Statistics 27. A *p*-value less than 0.05 was considered statistically significant. The graphical abstracts were created using Figdraw 2.0.

## 4. Conclusions

In the present study, we found that the peptide extracted from the nacre powder of *Hyriopsis cumingii* has a significant potential to inhibit tyrosinase. By LC-MS/MS and molecular docking screening, we identified the tyrosinase inhibitory peptide YPNPY from the pearl powder peptides; its IC_50_ value for tyrosinase was (0.545 ± 0.028) mM. The results of the inhibition kinetics showed that YPNPY was a reversible competitive inhibitor. Molecular docking and molecular dynamics simulations showed that YPNPY has a strong binding affinity with a binding energy of −10.4 kcal/mol. The effects of YPNPY on tyrosinase activity, melanogenesis, and antioxidant enzyme activities were investigated in mouse B16F10 melanoma cells. The results showed that YPNPY significantly inhibited intracellular tyrosinase activity and melanin content, and positively affected the intracellular antioxidant enzyme system. It can be concluded that the peptide YPNPY extracted from *Hyriopsis cumingii* has good therapeutic effects. This can be further confirmed by animal experiments and used in the development of food or cosmetics.

## Figures and Tables

**Table 1:** Potential tyrosinase inhibitory peptide sequences: screening by mass spectrometry and molecular docking.

No.	Peptide Sequence	Score	Molecular Weight	Length	Binding Energy /(kcal/mol)	GRAVY
1	YVPGHGW	35.00	814.4	7	−10.7	−0.51
2	GYHFHSYP	24.6	1006.4	8	−10.7	−1.13
3	YPNPY	24.27	652.3	5	−10.4	−1.86
4	YVTFTP	28.59	726.4	6	−9.5	0.45
5	YVPGHG	44.80	628.3	6	−9.4	−0.45
6	YFGGY	23.22	605.2	5	−9.3	−0.12
7	PVGGYY	21.99	654.3	6	−9.3	−0.13
8	EQYVPGH	36.93	828.4	7	−9.2	−1.33
9	GFPYGPG	22.72	693.3	7	−9.1	−0.41
10	EQYVPGHG	35.90	885.4	8	−9.0	−1.21
11	TVYTP	26.34	579.3	5	−9.0	−0.02
12	GHAVSTPRG	65.51	880.5	9	−8.9	−0.62
13	FDAQPDT	23.94	792.3	7	−8.9	−1.17

**Table 2:** Molecular docking sites of YPNPY, GYHFHSYP, and YVPGHG with tyrosinase (2Y9X).

Peptides	Hydrogen Bonds	Hydrophobic Interaction	Electrostatic Interaction
GYHFHSYP	HIS85 ALA246 GLY245 SER282 HIS244	PRO277 PRO284	-
YPNPY	HIS263 ASN260 ASN81	VAL283 ALA286	-
YVPGHG	ASN81 ALA323 ASN260 HIS244 VAL283	-	-
Kojic acid	HIS259 HIS263 MET280	-	

**Table 3:** Effects of AHYYD, TFSGNYP, KPIWT, and positive control Kojic acid on tyrosinase activity.

Substances	Peptide Sequence	Tyrosinase Inhibitory Activity IC_50_/(mM)
peptides	YPNPY	0.545 ± 0.028
	GYHFHSYP	>5
	YVPGHG	>50
Kojic acid	—	0.010 ± 0.003

## Data Availability

The data showed in this study are contained within the article.
